# Association Between the 5As and Stage of Change Among African American Smokers Eligible for Low-Dose Computed Tomography Screening

**DOI:** 10.5888/pcd18.210073

**Published:** 2021-07-15

**Authors:** Yu-Hsiang Kao, Tung-Sung Tseng, Michael D. Celestin, Jennifer Hart, Lucretia Young, Mirandy Li, Leonard R. Bok, David L. Smith, Jyotsna Fuloria, Sarah Moody-Thomas, Edward J. Trapido

**Affiliations:** 1 Behavioral and Community Health Sciences, LSUHSC School of Public Health, New Orleans, Louisiana; 2 Department of Medicine, LSUHSC School of Medicine, New Orleans, Louisiana; 3 Department of Radiology, LSUHSC School of Medicine, New Orleans, Louisiana; 4 University Medical Center, New Orleans, New Orleans, Louisiana; 5 Epidemiology, LSUHSC School of Public Health, New Orleans, Louisiana

## Abstract

We investigated the association between the 5As (Ask, Advise, Assess, Assist, and Arrange) clinical protocol and stage of change among African American smokers who are eligible for low-dose computed tomography screening. In 2019, 60 African American daily smokers aged 55 years or older were recruited in a large hospital in New Orleans, Louisiana. Smokers who received assistance for smoking cessation were more likely to be in the preparation stage than those who did not receive any assistance. Assistance from health professionals is an essential form of support and may substantially enhance smokers’ motivation to quit smoking in this population that is at higher risk for mortality from lung cancer.

**Summary**

**What is already known about this topic?**

The 5As (Ask, Advise, Assess, Assist, and Arrange) is a standard clinical protocol for treating tobacco use; smoking cessation quitting rates are associated with Assist and Arrange.

**What is added by this report?**

This study demonstrated that the Assist step is a critical component of enhancing motivation to quit among African American current smokers eligible for low-dose computed tomography (LDCT) screening.

**What are the implications for public health practice?**

Health professionals should assist African American smokers who are eligible for LDCT screening with quitting, including setting a specific quit date and using behavioral counseling to improve their motivations for smoking cessation.

## Objective

African Americans have higher rates of mortality from lung cancer compared with other racial/ethnic groups (1). Low-dose computed tomography (LDCT) screening can detect lung cancer early to decrease lung cancer–specific mortality (2) for smokers who are at high risk, but it remains underused in this population (3). Although LDCT screening and smoking cessation combined may result in higher quit rates (4), we know little about the effect of the 5As clinical protocol on readiness to quit for African American smokers who are eligible for LDCT screening. In this study, we investigated the association between the 5As and stage of change for this population that is at higher risk for mortality from lung cancer.

## Methods

In 2019, we recruited 60 African American daily smokers aged 55 years or older from primary clinics in a large hospital in New Orleans, Louisiana, who reported being told that they are eligible to receive LDCT screening. Enrollees completed a paper-based, anonymous survey that collected demographic information, tobacco use behaviors, and smoking cessation behaviors.

We used the question “Are you planning to quit smoking within the next 1 to 6 months?” to assess stage of change (5). Stages of change are precontemplation (no thought to quit or plan to quit within 6 months), contemplation (plan to quit within 6 months), and preparation (plan to quit within 1 month). The 5As is a standard clinical protocol for treating tobacco use (6); the 5As are 1) Ask about tobacco use (“In the past 12 months, did any health care provider at this hospital ask if you smoke?”); 2) Advise smokers to quit (“In the past 12 months, did any health care provider at this hospital advise you to quit smoking?”); 3) Assess smokers’ willingness to make a quit attempt (“In the past 12 months, when a health care provider at this hospital advised you to quit smoking did they also ask if you wanted to try to quit?”); 4) Assist smokers with making a quit attempt (“In the past 12 months, when a health care provider at this hospital advised you to quit smoking, did they do any of the following: (detailed list in Table 1)”; and 5) Arrange for follow-up contact (“Did a health care provider arrange for a counselor, program, or quitline to make follow-up contact with you?”).

**Table 1:** Association Between Assist and Stage of Change Using Multinominal Logistic Regression Models, African American Daily Smokers Aged 55 Years or Older (N = 59), Assessment of the 5As, New Orleans, Louisiana, 2019

Type of Assist	Precontemplation, n	Contemplation, n	Preparation, n	Contemplation vs Precontemplation, Crude OR (95% CI)	Preparation vs Precontemplation, Crude OR (95% CI)
Assist (any)^a^	5	19	19	3.8 (0.7–20.8)	15.2 (1.4–167.8)^b^
Prescribe or recommend a patch, nicotine gum, nasal spray, an inhaler, or pills such as Bupropion, Zyban, Wellbutrin, Varenicline, or Chantix	4	5	10	0.3 (0.1–1.8)	1.4 (0.3–6.8)
Suggest setting a specific date to stop smoking	1	8	11	4.3 (0.4–40.4)	11.0 (1.1–106.4)^b^
Suggest using a class, program, quit line, or counseling	2	18	17	12.6 (2.0–80.8)^c^	19.8 (2.7–145.7)^c^
Give booklets, videos, or other material to help quit	1	10	8	6.1 (0.7–57.6)	5.8 (0.6–56.3)

Abbreviation: 5As, Ask, Advise, Assess, Assist, and Arrange.

a A participant could receive one or more Assist options.

b *P* < .05.

c *P* < .01.

We used descriptive statistics to summarize participants’ demographics, nicotine dependence level (7), and responses to the 5As. We used multinominal logistic regression models to examine the association between the 5As and stage of change. Because we detected nonsignificant results between demographic characteristics and stage of change and because of the small sample size, we also conducted multinominal logistic regression models with a crude odds ratio (OR) for Assist. Significance for all tests was set at *P* < .05. All analyses were performed using SAS version 9.4 (SAS Institute). This study was reviewed and approved by the Institutional Review Board of Louisiana State University Health Sciences Center New Orleans.

## Results

Most participants were female (61.0%) and on average 61.1 years old (Table 2). Of the 59 participants who reported their stage of change, 25 (42.4%) smokers reported being in the preparation stage, 24 (40.7%) reported being in the contemplation stage, and 10 (16.9%) reported being in the precontemplation stage. The mean pack-year (calculated by multiplying the number of packs of cigarettes smoked per day by the number of years the person has smoked) history was 23.1 across all participants. Compared with the precontemplation stage, a higher percentage of smokers in the preparation stage were male, had lower income and education levels, were obese, and had a moderate addiction level. However, demographics, pack-year history, and nicotine addiction level did not show a significant association with stage of change. Regarding the 5As, more African American current smokers who are eligible for LDCT screening received the first 4 As (Ask [100%], Advise [90%], Assess [98%], and Assist [83%]), but only 29% received “Arrange.” Moreover, a significant difference was observed for “Assist” for quit attempts and stage of change (*P* = .04).

**Table 2:** Demographic Characteristics and Stage of Change^a^, African American Daily Smokers Aged 55 Years or Older (N = 59), Assessment of the 5As, New Orleans, Louisiana, 2019

Variable	Overall (N = 59)^b^	Precontemplation (n = 10)	Contemplation (n = 24)	Preparation (n = 25)	*P* Value^c^
**Demographic Characteristics**					
**Mean age, y (SD)**	61.1 (5.5)	60.7 (4.6)	60.9 (4.5)	61.4 (6.7)	.70^d^
**Sex**					
Female	36 (61.0)	7 (70.0)	15 (62.5)	14 (56.0)	.83
Male	23 (39.0)	3 (30.0)	9 (37.5)	11 (44.0)	
**Income, $**					
<20,000	54 (91.5)	9 (90.0)	20 (83.3)	25 (100.0)	.11
≥20,000	5 (8.5)	1 (10.0)	4 (16.7)	0	
**Education**					
≤Grade 12	24 (40.7)	3 (30.0)	8 (33.3)	13 (52.0)	.37
>Grade 12	35 (59.3)	7 (70.0)	16 (66.7)	12 (48.0)	
**Medicaid**					
No	17 (28.8)	2 (20.0)	8 (33.3)	7 (28.0)	.80
Yes	42 (71.2)	8 (80.0)	16 (66.7)	18 (72.0)	
**BMI, kg/m^2^**					
Normal weight	17 (28.8)	3 (30.0)	7 (29.2)	7 (28.0)	.09
Overweight	19 (32.2)	6 (60.0)	4 (16.7)	9 (36.0)	
Obese	23 (39.0)	1 (10.0)	13 (54.2)	9 (36.0)	
**Tobacco Use**					
**Pack year, mean (SD)**	23.1 (18.3)	31.0 (26.8)	21.8 (18.5)	21.1 (13.6)	.21^c^
**Nicotine dependence^e^**					
Very low and low	45 (76.3)	8 (80.0)	18 (75.0)	19 (76.0)	.81
Moderate	9 (15.3)	1 (10.0)	3 (12.5)	5 (20.0)	
High and very high	5 (8.4)	1 (10.0)	3 (12.5)	1 (4.0)	
**5As^f^**					
**Ask (missing, n = 1)**					
Yes	58 (100.0)	10 (100.0)	24 (100.0)	24 (100.0)	NA
**Advise (missing, n = 1)**					
No	6 (10.3)	1 (10.0)	1 (4.2)	4 (16.7)	.39
Yes	52 (89.7)	9 (90.0)	23 (95.8)	20 (83.3)	
**Assess^g^**					
No	1 (1.9)	0	0	1 (5.0)	.56
Yes	51 (98.1)	9 (100.0)	23 (100.0)	19 (95.0)	
**Assist^g^**					
No	9 (17.3)	4 (44.4)	4 (17.4)	1 (5.0)	.04
Yes	43 (82.7)	5 (55.6)	19 (82.6)	19 (95.0)	
**Arrange^g^**					
No	37 (71.2)	8 (88.9)	18 (78.3)	11 (55.0)	.14
Yes	15 (28.8)	1 (11.1)	5 (21.7)	9 (45.0)	

Abbreviations: 5As, Ask, Advise, Assess, Assist, and Arrange; BMI, body mass index; NA, not applicable; SD, standard deviation.

a Preparation: Plan to quit within 1 month; Contemplation: plan to quit within 6 months; Precontemplation: no thought to quit or plan to quit longer than 6 months.

b 59 African American smokers reported their stage of change. All values are no. (%) unless otherwise indicated.

c Determined by using Fisher Exact Test.

d Determined by using 1-way ANOVA.

e Heaviness of smoking index, which is a short version of the Fagerstrom test of nicotine dependence to identify patients’ nicotine dependence level, based on responses to the following 2 questions: 1) “On average, about how many cigarettes a day do you smoke now?” (scoring: less than 10 = 0; 10 through 19 =1; 20 through 29 = 2; and 30 or higher = 3); and 2) “How soon after you wake do you smoke your first cigarette?” (scoring: after 60 minutes = 0; from 31 to 60 minutes = 1; from 6 to 30 minutes = 2; within 5 minutes = 3). The total score ranges from 0 to 6 (0 to 2 = very low nicotine dependence; 3 = low nicotine dependence; 4 = moderate nicotine dependence; 5 = high nicotine dependence; 6 = very high nicotine dependence). Because of small sample sizes, we categorized nicotine dependence into 3 levels: very low and low (0–3), moderate (4), and high and very high (5–6).

f Each element of the 5As was determined by asking the following questions: 1) Ask: “In the past 12 months, did any health care provider at this hospital ask if you smoke?”; 2) Advise: “In the past 12 months, did any health care provider advise you to quit smoking?”; 3) Assess: “During the past 12 months, did any health care provider ask you if you were willing to make a quit attempt?”; 4) Assist: “In the past 12 months, when a health care provider advised you to quit smoking, did they do any of the following (such as prescribe or recommend medication, suggest setting quit date, recommend or refer to counseling, or give self-help material)?”; and 5) Arrange: “In the past 12 months, when a health care provider advised you to quit smoking, did they do any of the following (such as call and ask you about your quit attempt within one week or one month)?”

g Health care providers advised patient to quit smoking.

Smokers who received any assistance with quitting were more likely to be in the preparation stage (OR = 15.2; 95% CI, 1.4–167.8) compared with those who did not receive any assistance (Table 1). Smokers who were asked to set a specific date to stop smoking (OR = 11.0; 95% CI, 1.1–106.4) were more likely to be in the preparation stage versus the precontemplation stage. Smokers who got a recommendation to use a class, program, quitline, or counseling were more likely to be in the contemplation stage (OR = 12.6; 95% CI, 2.0–80.8) and the preparation stage (OR = 19.8; 95% CI, 2.7–145.7) than the precontemplation stage. 

## Discussion

A positive association between Assist and stage of change was evident among African American smokers eligible for LDCT screening. Although prior studies have shown that smokers who receive all 5 steps of the 5As intervention from health professionals are more likely to take action to quit smoking (8), many health professionals in the US do not routinely complete all 5 (9). Most health professionals provide the first 3 As (Ask, Advise, and Assess) (10); however, the percentage for delivering the final 2 As (Assist and Arrange) to African American smokers remains lower than among the White population (11). We found that most African American smokers who are eligible for LDCT screening received the first 4 As. Moreover, this population was more likely to be in the contemplation and preparation stages while receiving assistance from health professionals. Our findings are supported by a National Lung Screening Trial study that indicated that more intensive assistance interventions may urge this population that is at higher risk for mortality from lung cancer to take action to quit smoking (12).

This study has limitations. First, this study is cross-sectional and used self-reported data, which tends to give narrow estimated associations. Second, a small sample size from a single hospital limits the generalizability. Lastly, Ask, Advise, and Assess were unable to provide robust estimations due to sample size limitations. Despite these limitations, our results suggest that African American smokers who are eligible for LDCT screening may be in the contemplation and preparation stage of quitting and should be offered quit assistance. Additionally, results indicated that Assist is an important component of enhancing quit intention for this population. Quit smoking assistance and LDCT screening are both important approaches for lung cancer prevention and control. To improve smoking cessation for this population, cessation programs and health professionals are encouraged to provide assistance during these opportune times.
